# The Magazines of the Medical Schools

**Published:** 1921-03-19

**Authors:** 


### The Magazines of the Medical Schools.

ST. THOMAS'S HOSPITAL GAZETTE.

THE centenary meeting of the Medical and Physical Society was preceded by a dinner in the Students' Club. When the company had adjourned to the Governors' Hall, Professor Mellanby gave a *résumé* of eminent physiologists of the hospital between 1820 and 1920. Subsequent speakers were Professor Parsons, who described the life and works of George Raimey, the anatomist, and Dr. Corner, who spoke of the twenty-four men who had served on the surgical staff since 1820. Dr. Turney told a good story of a certain Dr. S., who on one occasion was endeavouring to obtain a specific history from a woman who stoutly denied all venereal disease. Finally, after prolonged ill-success, he said : " How dc you cook a bullock's heart ? " The patient did not know. " Every *respectable* woman knows how to cook a bullock's heart, he said, and triumphantly put her on antisyphilitic treatment at once.

GUY'S HOSPITAL GAZETTE.

The March number contains a fine portrait of John Keats, and a republished article gives a pleasing and instructive account of the years that the poet spent at Guy's, where he was known by his fellow-dressers as "Tiny Keats." While at Guy's, Keats wrote and composed several of his shorter pieces, all of which giving promise of the marvellous development that was to come during the few years immediately preceding his death. That wonderful line,

" Silent upon a peak of Darien,"

was written before he came to the hospital, while the exquisitely finished ballad,

" Oh ! what can ail thee, knight at arms,
Alone and palely loitering ? "

is perhaps the only one of his better-known poems that was certainly finished in his hospital days.

ST. MARY'S HOSPITAL GAZETTE.

The February issue sounds a justifiably satisfied note on the theme of the successful exhibit of the hospital at the Efficiency Exhibition at Olympia, and claims that any man who inspected the hospital stall and listened to the explanations so clearly given by seniors and juniors must have been awakened to the wideness of the field of medical science, and have obtained some glimpses of the far-off horizon towards which science is moving. It is claimed that by this venture St. Mary's Hospital has done something of real value to impress upon the lay public the overwhelming importance of research, and it should help the appeal for funds for the improvement of the school buildings and for the endowment of a chair and laboratory of industrial medicine.

ST. BARTHOLOMEW'S HOSPITAL JOURNAL.

The March number has an excellent account of the laying of the foundation-stone of the Nurses' Home by the Queen. There is also an amusing forecast of what Bart's may be like in 2195, an era when the minimum period required for medical qualification will be twenty-seven years. Each of the seventeen stories of the medical school building has its wonders. In the physics school, to select one part of the vast establishment, one finds the laboratories of electro-pyscho-analysis, æthero-dynamics, lunar acoustics, asteroid epidemiology, wireless telepathy, and aura-photography. In the Einsteinian Laboratory of Relativity Professor McWo is found engaged in a demonstration of the parallelopiped of warped forces. An ingenious combined microtome and section-stainer, " resembling in appearance a large calculating machine," must save much labour in the Department of Clinical Pathology, where also is found an instantaneous sputometer, which has but to be spat into to record within five seconds the presence or absence of the elusive tubercle bacillus.

